# Facial Baroparesis Caused by Scuba Diving

**DOI:** 10.1155/2012/329536

**Published:** 2012-02-19

**Authors:** Daisuke Kamide, Takeshi Matsunobu, Akihiro Shiotani

**Affiliations:** ^1^Division of Otolaryngology, Japan Self Defense Force Hospital Kure, Hiroshima 737-0027, Japan; ^2^Department of Otolaryngology-Head and Neck Surgery, National Defense Medical College, Saitama 359-8513, Japan

## Abstract

Middle ear barotrauma is one of the common complications of SCUBA diving representing acute otalgia, hearing loss, and bleeding. But occurrence of facial palsy is rare. Here we report a case of a 30-year-old navy diver suffered middle ear barotrauma with transient facial palsy after SCUBA diving. He felt difficulty in equalizing the pressure in middle ear with Valsalva maneuver during diving, and suffered right facial palsy and aural fullness after diving. Clinical examination showed remarkable bulging of the right tympanic membrane and right facial palsy without other neurological findings. But facial palsy was disappeared immediately after myringotomy. We considered that the etiology of this case was neuropraxia of facial nerve in middle ear caused by over pressure of middle ear.

## 1. Introduction

Scuba diving is nowadays sgetting popular for both occupational and recreational purposes. As a rapid pressure change in the middle ear makes it liable to problems during descent or ascent, middle ear overpressure or barotrauma is common complication of diving which represents acute otalgia, hearing loss, and bleeding. However, facial nerve palsy (facial baroparesis) is extremely rare. Now we report a case of facial palsy caused by barotraumas of middle ear after diving and cured by simple myringotomy completely. This is a case without precedent in the world literature.

## 2. Case Report

In June 2007, a 30-year-old navy diver joined a training program on scuba diving, in which the maximum depth of each dive was approximately 8 meters. Before the training, he experienced difficulty in equalizing the pressure in his ears with the surrounding water pressure, but he continued diving. During descent, he repeatedly performed the Valsalva maneuver to equalize the pressure in his ears. He experienced right aural fullness and pain in the right ear during ascent and right facial numbness and hearing loss in his right ear shortly after surfacing. He then visited our naval hospital on foot. Neurological examination revealed facial palsy (House-Brackmann Grade IV) and mixed hearing loss in the right ear. Remarkable bulging of the right tympanic membrane was observed, but no bleeding, tearing, and congestion were noted. No vestibular disturbance was observed with him.

**Figure 1:**
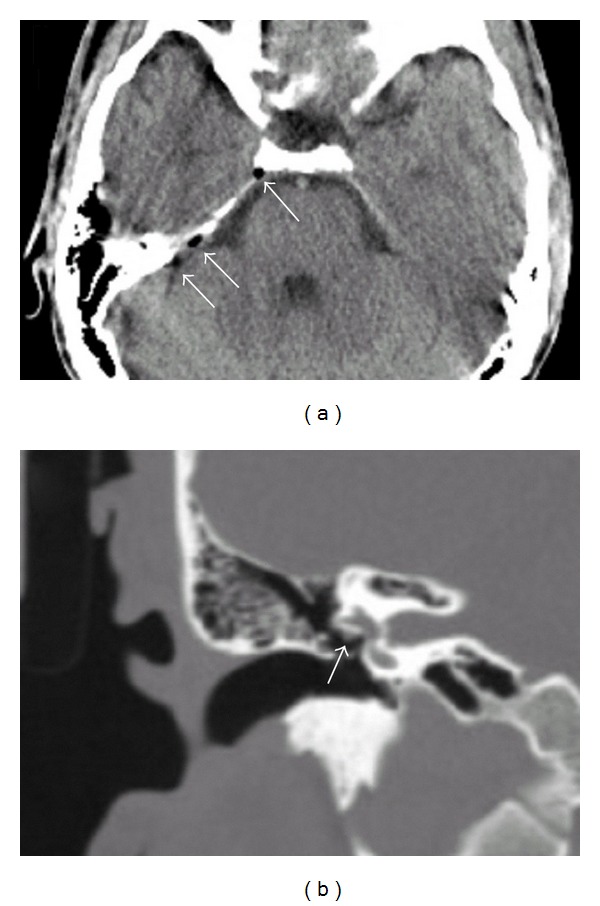
(a) Brain CT (axial section) free air (arrow) is observed in the subdural space. (b) Temporal bone target CT (sagittal section); the tympanic segment of the facial nerve (arrow) appears to be directly exposed to the tympanic cavity.

Brain computed tomography (CT) revealed free air in the right subdural space in the midcranial fossa (Figure 1(a)). Since the neurological symptoms developed after diving, we considered the possibility of decompression sickness. Therefore, we attempted to treat him using a recompression chamber. Before recompression, we performed myringotomy of the right tympanic membrane to equalize the pressure in the middle ear with the atmospheric pressure. Immediately after the myringotomy, a hissing sound was heard. Surprisingly, at the same time, the facial palsy, numbness, and hearing impairment almost completely disappeared (House-Brackmann Grade I). He returned to work duty without any residual symptoms after a week.

## 3. Discussion

The middle ear is an air-filled space separated from the external environment by the tympanic membrane. The pressure in the middle ear is equalized via the Eustachian tube, which opens into the nasopharynx. Eustachian tube function can be easily impaired by mucosal inflammation caused by common cold, allergic rhinitis, and bronchitis. The typical symptoms of middle ear barotrauma are acute otalgia, hearing loss, and bleeding in the middle ear; however, facial nerve palsy is rare [1]. 

The cause of facial palsy can be explained by its anatomical characteristics. The facial nerve enters the temporal bone through the internal auditory canal from the brainstem. The tympanic segment of the facial nerve is surrounded by a very thin bony wall in the middle ear cavity and its mastoid segment, by the mastoid air cells in the temporal bone. Some studies have revealed that because of the anatomical characteristics, the bony canal of facial nerve can undergo spontaneous dehiscence. A large study of temporal bone showed that dehiscence of the facial nerve canal was found in 55% of normal adult temporal bones [2]. In this case, the apparent exposure of the facial nerve in the tympanic cavity was observed on a CT scan (Figure 1(b)). And as described in the study using animal models, increased pressure in the middle ear, which exceeds the capillary blood pressure, decreases the blood supply to the facial nerve and induces ischemic neurapraxia because of canal dehiscence [3]. Decompression sickness is the major differential diagnosis of neurological disorders that develop shortly after diving. Because facial palsy occurred soon after surfacing and rapidly disappeared after the removal of the air by means of a myringotomy, the mechanism of facial palsy in this case is increased pressure in the tympanic cavity due to the performance of the Valsalva maneuver in the presence of Eustachian tube malfunction.

Intracranial air (pneumocephalus) in this case could be caused by the trapped air in the middle ear by Valsalva maneuver and went into the subdural space through the thin bony wall of tegmen tympani [4] or the inner auditory canal. Because small free air can be absorbed spontaneously, no additional surgical treatment was needed in this case.

The slight sensorineural hearing loss may have been due to transient inner ear barotrauma caused by increased pressure in the tympanic cavity or in the internal auditory canal.

A misdiagnosis of severe decompression sickness may threaten the ability of professional divers to pursue their profession. Facial palsy caused by increase pressure in the middle ear is transient, provided that appropriate treatment, that is, myringotomy, is administered. This treatment is entirely different from the treatment for decompression sickness. Simply observing the tympanic membrane could be important for both the diagnosis and treatment of diver's facial palsy.

In conclusion, overpressure in the middle ear induces facial nerve neuropraxia. Careful interview of the history and examination of tympanic membrane is important to avoid unnecessary and long burden of the recompression treatment. Furthermore immediate myringotomy could be a powerful treatment against facial palsy caused by barotrauma in the middle ear.
